# Typing of *Ochrobactrum anthropi* clinical isolates using automated repetitive extragenic palindromic-polymerase chain reaction DNA fingerprinting and matrix-assisted laser desorption/ionization–time-of-flight mass spectrometry

**DOI:** 10.1186/1471-2180-14-74

**Published:** 2014-03-22

**Authors:** Angela Quirino, Giovanna Pulcrano, Linda Rametti, Rossana Puccio, Nadia Marascio, Maria Rosaria Catania, Giovanni Matera, Maria Carla Liberto, Alfredo Focà

**Affiliations:** 1 Institute of Microbiology, Department of Health Sciences, “Magna Graecia” University, Viale Europa, Catanzaro, Italy; 2 Department of Molecular and Cellular Biology and Pathology L. Califano, Medicine School, University of Naples Federico II, Naples, Italy

**Keywords:** *Ochrobactrum anthropi*, rep-PCR fingerprinting, MALDI-TOF MS, Strain typing

## Abstract

**Background:**

*Ochrobactrum anthropi* (*O. anthropi*), is a non-fermenting gram-negative bacillus usually found in the environment. Nevertheless, during the past decade it has been identified as pathogenic to immunocompromised patients. In this study, we assessed the usefulness of the automated repetitive extragenic palindromic-polymerase chain reaction (rep-PCR-based DiversiLab™ system, bioMèrieux, France) and of matrix-assisted laser desorption/ionization-time-of-flight (MALDI-TOF MS) for typing of twentythree *O. anthropi* clinical isolates that we found over a four-months period (from April 2011 to August 2011) in bacteriemic patients admitted in the same operative unit of our hospital. Pulsed-field gel electrophoresis (PFGE), commonly accepted as the gold standard technique for typing, was also used. Analysis was carried out using the Pearson correlation coefficient to determine the distance matrice and the unweighted pair group method with arithmetic mean (UPGMA) to generate dendogram.

**Results:**

Rep-PCR analysis identified four different patterns: three that clustered together with 97% or more pattern similarity, and one whose members showed < 95% pattern similarity. Interestingly, strains isolated later (from 11/06/2011 to 24/08/2011) displayed a pattern with 99% similarity. MALDI-TOF MS evaluation clustered the twentythree strains of *O. anthropi* into a single group containing four distinct subgroups, each comprising the majority of strains clustering below 5 distance levels, indicating a high similarity between the isolates.

**Conclusions:**

Our results indicate that these isolates are clonally-related and the methods used afforded a valuable contribution to the epidemiology, prevention and control of the infections caused by this pathogen.

## Background

*Ochrobactrum anthropi* (*O. anthropi*) is a non-fermenting, aerobic, gram-negative bacillus that exhibits widespread resistance to β-lactam antibiotics [1,2] and is able to colonize a variety of environments, namely soil, plants, insects, animals and humans [3]. Reports of opportunistic/nosocomial infections caused by *O. anthropi* have been increasing over the last decade [4-6], and the ability of *O. anthropi* to adhere to silicone may play a role in catheter-associated infections [6,7]. Furthermore, *O. anthropi* populations may adapt in response to habitat and host interactions, as previously described in human clinical isolates [3,8]. In the human infection: a catheter-associated bacteremia caused by *O. anthropi* has been shown [1]. In literature, the infections due to *O. anthropi* involved catheter related bacteremia, whereas endophalmitis, urinary infections, meningitis, endocarditis, hepatic, pelvic and pancreatic abscess often as monomicrobial infection have been reported [1,4,6,9]

According to their habitat, the population structure of *O. anthropi* varied. For example, biological and genomic microdiversity was higher in bulk soil than in the rhizoshere [10,3]. Authors related this difference in diversity level to the expansion of clones adapted to metabolites produced by rhizodeposition [3].

Among the few publications regarding the known methods for typing of *O. anthropi* relevant papers are those from Romano et al., 2010 [3] dealing with MLST and PFGE. Also, Bathe et al., 2006 [11] described the rep-PCR of *O. anthropi* (however with a instrument different than Diversilab, bioMerieux). Finally, Bizzini et al., 2010 [12] reported on Maldi-TOF characterization of *O. anthropi.*

The different typing methods used, mainly rep-PCR and Maldi-TOF, in terms of time, accuracy and costs may allow to obtain more timely, accurate results with higher resolution among the different strains involved in hospital outbreak. When this infection did occur in our hospital, we set out to study the identification and typing of the twentythree *O. anthropi* strains. Strain typing was carried out by automated repetitive extragenic palindromic-polymerase chain reaction (rep-PCR-based DiversiLab^TM^ system, bioMèrieux, France) and by pulsed-field gel electrophoresis (PFGE). Proteome profiling was performed through matrix-assisted laser desorption/ionization-time-of-flight (MALDI-TOF MS).

The application of accurate and more powerful techniques, used for typing, should be encouraged for monitoring the spread of bacteria and nosocomial infection control.

## Methods

### Bacterial strains and microbiological methods

During a 4-month period (from April 2011 to August 2011) 23 *O. anthropi* strains were isolated from samples of 19 patients admitted to the Catanzaro University Hospital (Italy) Oncology O.U. Samples were taken as part of standard patient care and all procedures were approved by the local ethics committee at the Medical Faculty of the University “Magna Graecia” of Catanzaro, which are in compliance with Declaration of Helsinki (59th WMA General Assembly, Seoul, October 2008).

During stay in hospital, all patients, which presented severe background disease, mainly neoplasia, showed mild clinical signs of sepsis. We therefore performed blood cultures by BacT/Alert 3D system (bioMèrieux, Clinical Diagnostics, France), detecting 18 isolates from 18 positive blood cultures drawn from the central venous catheter (CVC) and 5 isolates from positive catheter tip cultures (Table 1). The strains were conventionally identified by typical Gram stain morphology and biochemical testing (Vitek-2, bioMèrieux, France). Antibiotic sensitivity was evaluated by Vitek System (bioMèrieux, France). To exclude Brucella misdiagnosis, the *O. anthropi* colonies of all isolates were tested with *Brucella* agglutinating sera (*Brucella spp*., *Brucella abortus* and *Brucella melitensis*).

**Table 1:** ***O. anthropi *** **strains isolated from patients admitted to the Oncology O.U.**

**Strain ID**	**Patient ID**	**Isolation location**	**Date of isolation**
**CZ1403**	**1**	**Blood**	**26/04/2011**
CZ1424*	**2**	**Blood**	**17/05/2011**
CZ1427*	**3**	**Blood**	**19/05/2011**
**CZ1425**	**4**	**Blood**	**20/05/2011**
CZ1429*	**3**	**Catheter tip**	**25/05/2011**
**CZ1433**	**5**	**Blood**	**06/06/2011**
**CZ1439**	**6**	**Blood**	**06/06/2011**
**CZ1442**	**7**	**Blood**	**09/06/2011**
CZ1443*	**2**	**Catheter tip**	**09/06/2011**
CZ1449*	**3**	**Catheter tip**	**11/06/2011**
**CZ1454**	**8**	**Blood**	**17/06/2011**
**CZ1458**	**9**	**Blood**	**20/06/2011**
**CZ1460**	**10**	**Blood**	**21/06/2011**
**CZ1474**	**11**	**Blood**	**29/06/2011**
**CZ1476**	**12**	**Blood**	**29/06/2011**
**CZ1505**	**13**	**Catheter tip**	**07/07/2011**
CZ1504*	**14**	**Blood**	**08/07/2011**
CZ1523*	**14**	**Catheter tip**	**14/07/2011**
**CZ1532**	**15**	**Blood**	**15/07/2011**
**CZ1519**	**16**	**Blood**	**19/07/2011**
**CZ1541**	**17**	**Blood**	**20/07/2011**
**CZ1552**	**18**	**Blood**	**26/07/2011**
**CZ1573**	**19**	**Blood**	**24/08/2011**

*strains isolated from the same patient.

### Rep-PCR-based DNA fingerprinting by the DiversiLab System

For rep-PCR analysis, bacteria (23 clinical strains of *O. anthropi*, in addition to *O. anthropi* ATCC49188T and *O. intermedium* LMG3301T, kind gifts from Dr. Fabien Aujoulat, Universitè Montpellier, France) were grown on Columbia blood agar; DNA was extracted from a 10-μl loopful of each *O. anthropi* colony, using an UltraClean Microbial DNA isolation kit (Mo Bio Laboratories, Carlsbad, CA). The extracted DNA was amplified using a DiversiLab Generic DNA fingerprinting kit (bioMèrieux, France), following the manufacturer’s instructions. DiversiLab Rep-PCR was performed according to Treviño M. et al., 2011 [13]. Briefly, 50 ng of genomic DNA, 2.5 U of AmpliTaq DNA polymerase, and 1.5 μl of 10× PCR buffer (Applied Biosystems, Foster City, CA) were added to the appropriate rep-PCR master mix to achieve a total of 25 μl. Thermal cycling parameters were as follows: initial denaturation at 94°C for 2 min, 35 cycles of denaturation at 94°C for 30 s, annealing at 60°C for 30 s, extension at 70°C for 90 s, and final extension at 70°C for 3 min. DNA concentration was measured by NANODROP 1000 Spectrophotometer (Thermo Scientific). The amplified product was stored at -20°C until detection. Analysis of rep-PCR products was performed using a DiversiLab system, in which the amplified fragments of various sizes and intensities are separated and detected using a microfluidic LabChip by an Agilent 2100 Bioanalyzer (Agilent Technologies, Palo Alto, CA). The relatedness of the isolates was analysed by means of the DiversiLab software, version 3.4, using the Pearson correlation coefficient, to determine distance matrices, and the unweighted-pair group method with arithmetic mean (UPGMA). Further analysis was performed by the Kullback–Leibler distance correlation coefficient.

In general, “different” was defined as <95% pattern similarity and 2 or more band differences for organisms defined as homogeneous, and three or more band differences for organisms defined as heterogeneous. “Similar” was defined as >95% and <97.% pattern similarity and one band difference for homogeneous organisms, or up to two band differences for heterogeneous organisms. “Indistinguishable” was defined as >97% pattern similarity and no band differences, including no variation in the intensities of individual bands, although overall intensities may differ [13].

### Genotyping by PFGE

The 23 *O. anthropi* isolates were grown overnight and suspended in SE buffer (75 mM NaCl, 25 mM EDTA, pH 7.5). The cell suspensions (4 McFarland units) were mixed with an equal volume of 2% low-melting point agarose, moulded into plugs at 4°C, and lysed with lysis buffer (1% N-lauryl sarcosine, 0.5 M EDTA, pH 8) supplemented with Proteinase K (500 μg/mL). The DNA contained in each plug was digested by 20 U of *SpeI* restriction enzyme in accordance with the manufacturer's instructions. Macrorestriction fragments were separated using the CHEF-DR III PFGE system (Bio-Rad) at 10°C for 20 h, at a field strength of 6 V/cm, with an initial switch time of 5 s and a final switch time of 35 s. A lambda ladder of phage DNA concatemers was used as a size marker. *O. anthropi* ATCC 49188 T and *O. intermedium* LMG 3301 T were also genotyped by PFGE, and fragment patterns were compared according to the criteria described by Tenover [14].

### MALDI-TOF MS and data analysis

A single colony grown overnight on TSI (Triple Sugar Iron) agar was spotted in duplicate onto the MALDI target (MSP 96 target polished steel (MicroScoutTarget) plate; (Bruker Daltonik, Bremen, Germany) and air-dried at room temperature. After air-drying, each spot was overlaid with 1 μL of HCCA (a-cyano-4-hydroxycinnamic acid) matrix solution saturated with organic solvent (50% acetonitrile and 2.5% trifluoroacetic acid) and air-dried completely before MALDI-TOF MS. MALDI-TOF MS was carried out using a MALDI Microflex LT. Peptide mass fingerprint product ion spectra were acquired in linear positive mode at a laser frequency of 20 Hz, within a mass range from 2000 to 20 000 Da. For each spectrum, 240 laser shots were automatically acquired in 40 shot steps from different positions of the target spot (random walk movement) using AutoXecute acquisition control software (Flexcontrol 3.0; Bruker Daltonics, Bremen, Germany). The spectra were externally calibrated using the standard calibrant mixture (*Escherichia coli* extracts supplemented by proteins RNase A and myoglobin; Bruker Daltonics). To identify unknown bacteria, each peak list generated was matched directly against reference libraries (3502 species). Unknown spectra were compared with a library of reference spectra by means of a pattern-recognition algorithm making use of peak position, peak intensity distributions and peak frequencies. MALDI-TOF identifications were classified using modified versions of the score values proposed by the manufacturer: a score ≥2 indicated species identification, a score in the range 1.7-1.99 indicated genus identification, and a score <1.7 denotes no identification. For the phylogenetic data analysis, a total of 16 spectra were automatically acquired with the AutoXecute acquisition control software for each strain (biological and technical replicates). MSP creation was carried out with the default setting of the Biotyper software (desired mass error for the MSP: 200; desired peak frequency minimum: 25%; maximum desired peak number for the MSP: 70). Each Minimum spanning trees (MSP) was assigned to its specific node on the taxonomy tree. In order to visualize the relationship between the MSPs, dendrogram clustering was carried out using the standard settings of MALDI Biotyper software version 2.0 (distance measure: correlation; linkage: average). In addition, to evaluate the spectral variation within each strain, the composite correlation index (CCI) was computed by loading the raw data into the Biotyper software [15].

## Results

### Phenotype analysis

All isolated strains exhibited the same biochemical pattern (excellent identification: 99%) and presented an overlapping antimicrobial susceptibility profile – they were all sensitive to gentamicin (<1 μg/ml), tobramycin (<1 μg/ml), amikacin (16 μg/ml), ciprofloxacin (<0.25 μg/ml), levofloxacin (0.25 μg/ml), imipenem (2 μg/ml), and sulfamethoxazole/trimethoprim (<20 μg/ml), and resistant to ampicillin (>32 μg/ml), ampicillin/sulbactam (>32 μg/ml), cefazolin (>64 μg/ml), cefepime (>64 μg/ml), cefoxitine (>64 μg/ml), ceftazidime (>64 μg/ml), ceftriaxone (>64 μg/ml), piperacillin/tazobactam (>128 μg/ml) and nitrofurantoin (256 μg/ml). The negative *Brucella* agglutination sera test supported the biochemical identification.

### Rep-PCR-based DNA fingerprinting

**Figure 1:**
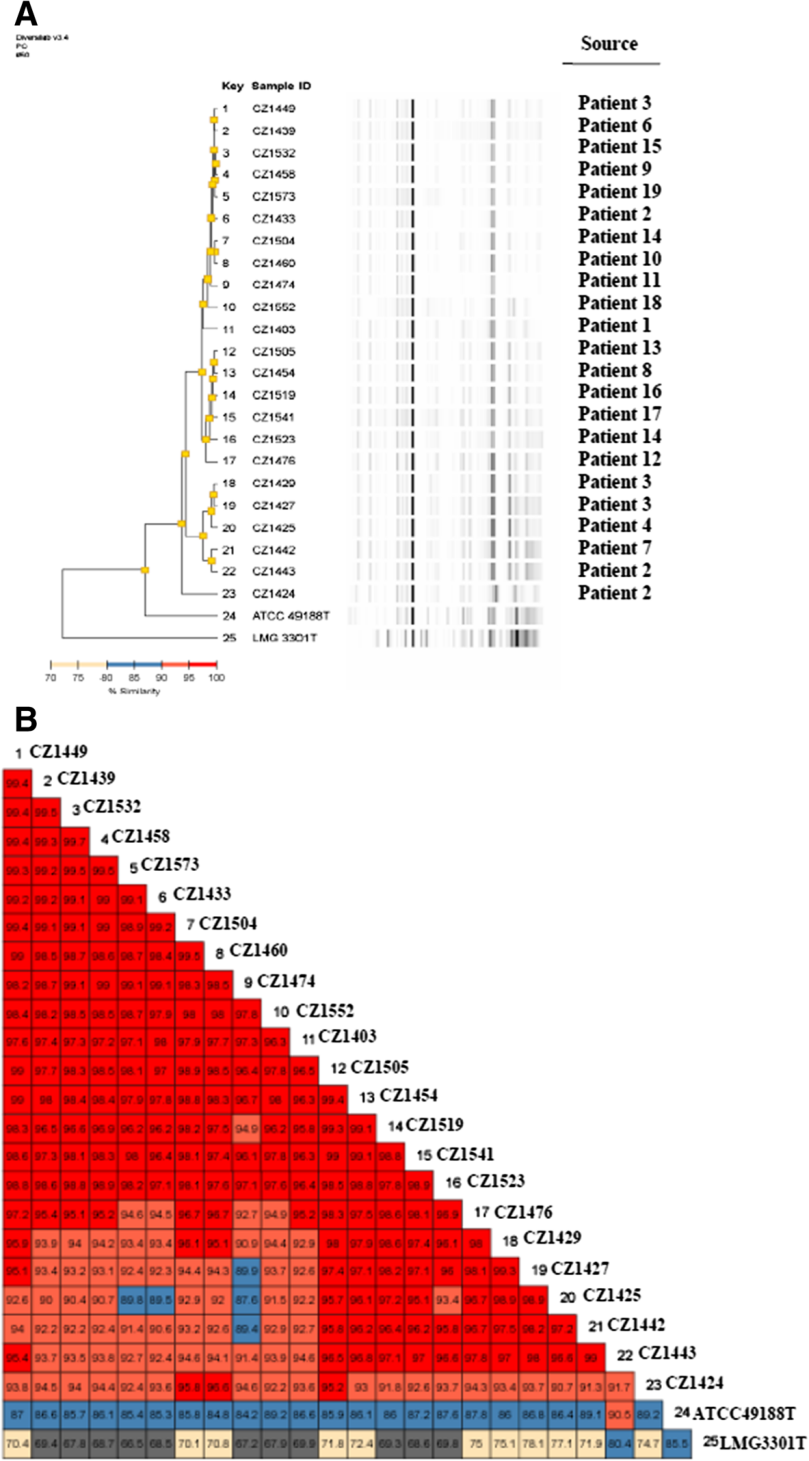
**Dendrogram, virtual gel image (panel A) and similarity matrix (panel B) of 23 *Ochrobactrum anthropi *strains, *O. anthropi *ATCC 49188 T and *O. intermedium *LMG 3301 T, investigated by the DiversiLab System and further analyzed by Pearson correlation.** (In Panel B the different colours and colour intensity refer to percentage of similarity).

Genome fingerprinting was carried out using the DiversiLab System (bioMèrieux, France), which uses two interpretation criteria: Pearson correlation, based on the intensity of electrophoretic bands, and Kullbach correlation, focused on the height and width of the peaks. Rep-PCR analysis identified four different patterns, as shown in the dendrogram in Figure 1 (panel A). Three rep-PCR patterns clustered isolates with 97% or more pattern similarity, and a further strain, CZ1424, showed a pattern of similarity of < 95%. This strain showed a correlation index of 91.7% when compared with strain CZ1443, isolated from a different site in the same patient. Pearson correlation, associated to chronological evaluation of the clinical isolates, showed that strains found during the first timespan (from 26/04/2011 to 09/06/2011 as shown in Table 1) exhibited an overlap between 90 and 99%, and were included in two different clusters (b and c). During the following timespan, up to the date of last bacterial isolation (24-08-2011), strain similarity was higher than 99%; accordingly these bacteria were grouped in a single cluster (a). Unlike strains CZ1424 and CZ1443, bacterial strains isolated from the same patients from two different sites were similar or indistinguishable when their genome fingerprints were compared. In particular, CZ1427 and CZ1429 strains overlap by 99%, CZ1429 and CZ1449 by 96% and CZ1427 and 1449 by 95.1%. A similar behaviour was noted between strains CZ1504 and CZ1523 (98.1% overlap) (Figure 1, panel B). In addition, as illustrated in Figure 1, panel B, all clinical strains investigated showed a pattern of similarity lower than 90.5% and 80.4% when compared to *O. anthropi* ATCC 49188 T and *O. intermedium* LMG 3301 T respectively. Kullback–Leibler analysis showed that the strains obtained later on in the outbreak, particularly 40 days after the first isolation, presented an inter-correlation greater than 92% (data not shown).

### PFGE data

**Figure 2:**
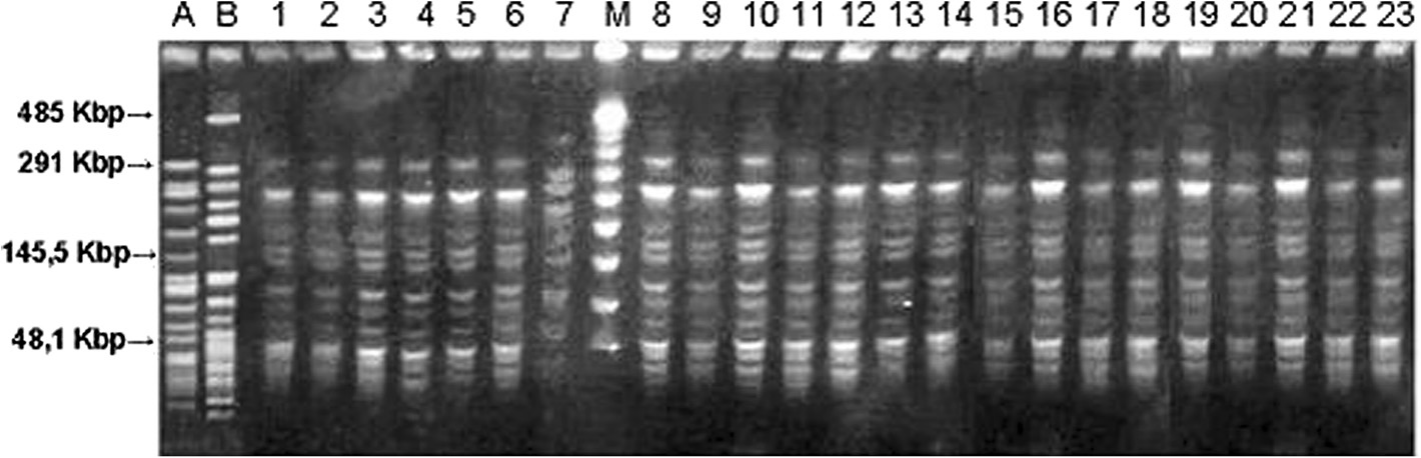
**PFGE analysis of *Ochrobactrum anthropi *** **ATCC 49188 T (A), *Ochrobactrum intermedium LMG 3301 T *** **(B) and of 23 *Ochrobactrum anthropi *** **strains: (1)CZ1425,(2)CZ1424, (3)CZ1460, (4)CZ1442,(5)CZ1443,(6)CZ1449,(7)CZ1552,(M)molecular weight marker,(8)CZ1433,(9)CZ1504,(10)CZ1523,(11)CZ1427,(12)CZ1532(13)CZ1541,(14)CZ1439,(15)CZ1454,(16)CZ1519,(17)CZ1458,(18)CZ1476,(19)CZ1403, (20)CZ1573,(21)CZ1505,(22)CZ1474,(23)CZ1429.**

The 23 strains of *O. anthropi* were typed by digestion of the chromosomal DNA with *SpeI* endonuclease, and fragment separation was obtained by PFGE. Each pattern consisted of approximately 10–15 fragments, which were found to be identical to each other, except for strain CZ 1552, whose 10–15-fragment pattern featured 6–7 fragment differences respect to the other pattern in the region between 145.5 and 485 Kbp. PFGE analysis thereby detected 22/23 unique pulsotypes with a high degree of inter-relatedness. *O. anthropi* ATCC 49188 T and *O. intermedium* LMG 3301 T appeared different from the 23 clinical isolates when compared according to Tenover’s criteria (Figure 2).

### MALDI-TOF MS data

**Figure 3:**
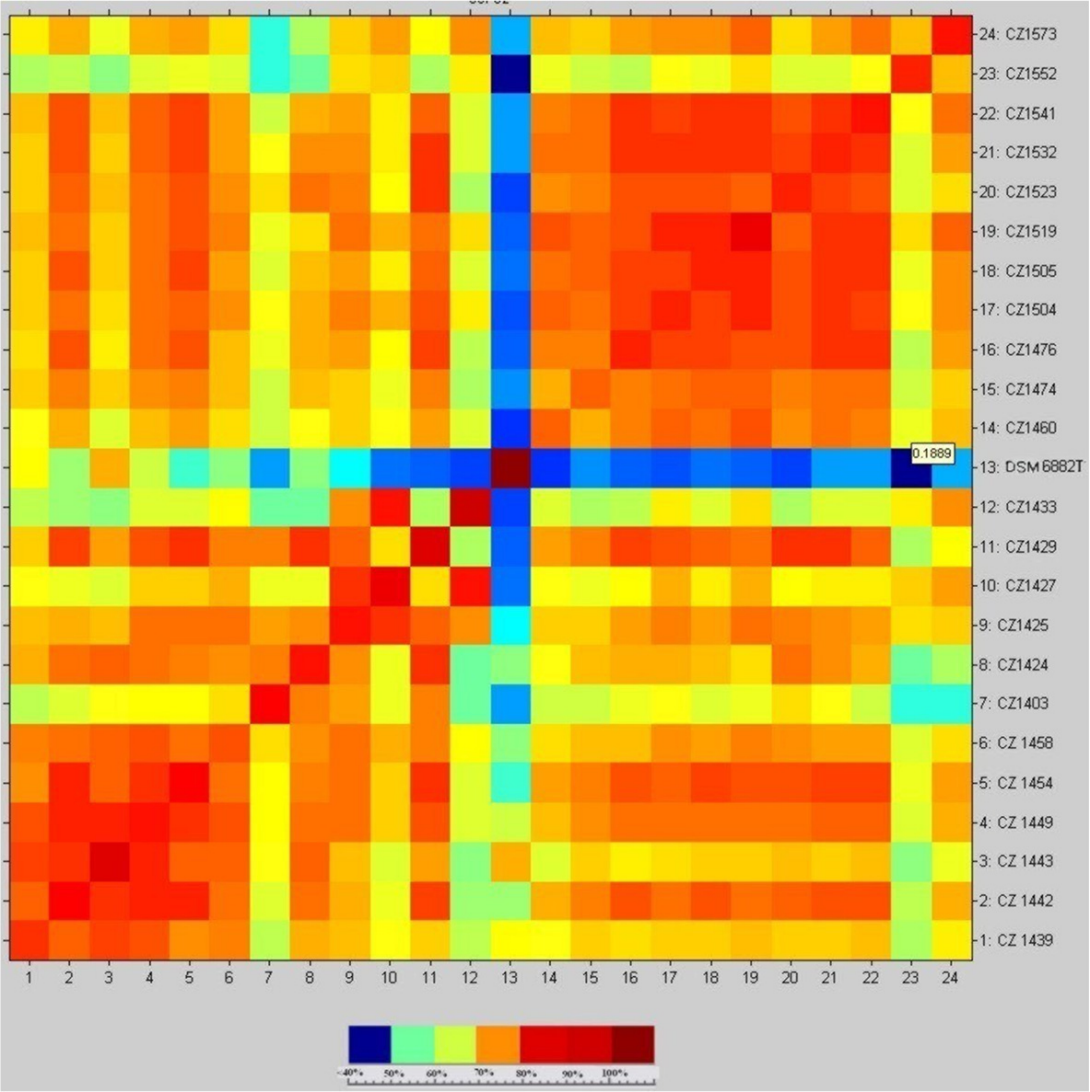
**Composite correlation index (CCI) matrix value for the strains of *Ochrobactrum anthropi.*** Different colors indicate the correlation distance. CCI was calculated with MALDI Biotyper 2.0 software at the default settings: the lower boundary is 2000, the upper boundary is 20,000, the resolution of the mass range is four, and the number of intervals for CCI is four. A CCI value near 1.0 indicates relatedness between the spectral sets, and 0.02 indicates the lowest match.

A total of 46 spectra representing the 23 strains of *O. anthropi* were generated with the automated MALDI-TOF MS measurement. Protein mass patterns were detected in the mass range 2000–20,000 Da, were matched against Bruker Daltonics reference library, which included three *O. anthropi* ATCC strains, and resulted correctly identified at the species level (log score ≥ 2). In order to create reliable MSPs for phylogenetic analysis, we measured a total of 368 spectra, 16 for each strain. Each mass spectrum dataset was compared with the others, yielding a matrix of cross-wise relatedness computed with the default setting provided by Biotyper 2.0 (CCI matrix). A CCI value approaching 1.0 showed confirmation of the set of spectra at a high level of significance, and is shown in Figure 3 by the brown squares at the diagonal intersection of the samples (maximum = self-to-self correlation). Inter-sample comparisons showed decreasing colour to yellow–blue, corresponding to decreasing degrees of correlation down to 0.02, the lowest match. Composite correlation index analysis for the 23 *Ochrobactrum anthropi* strains showed similar inter-strain relatedness (Figure 3). Strains CZ1424 and CZ1443, as well as strains CZ1523 and CZ1504, isolated from the same patients but from two different sites, shared high degrees of similarity (over 80% and 85% respectively). Lower similarity, ranging from 60 to 80%, was found among strains CZ1427, CZ1429 and CZ1449, also isolated from two different sites in the same patient. Strains CZ 1403, CZ1433 and CZ1442 showed the lowest degree of similarity with other strains (less than 20%). At the other end of the scale, two strain clusters (CZ1439, CZ1442, CZ1443, CZ1449, CZ1454, CZ1458 and CZ1460, CZ1474, CZ1476, CZ1504, CZ1505, CZ1519, CZ1523, CZ1532, CZ1541) shared a high degree of similarity (up to 95%).

**Figure 4:**
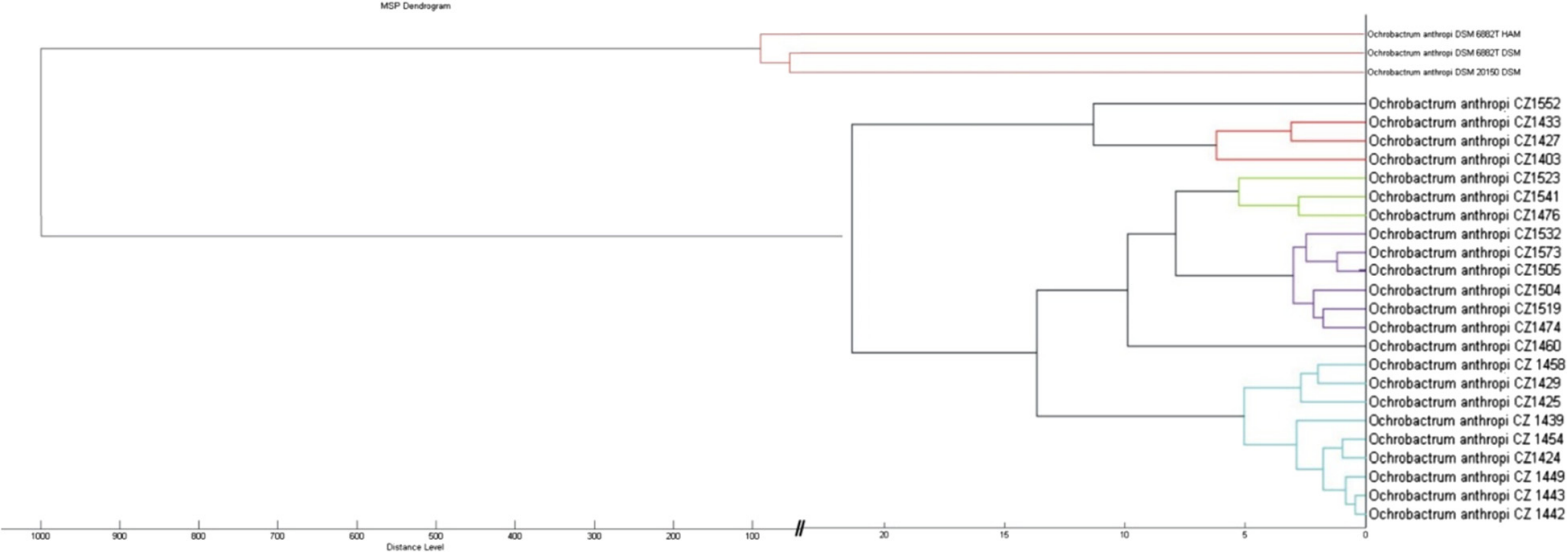
Score-oriented dendrogram of matrix-assisted laser desorption ionization time-of-flight mass spectrometry profiles generated by the default setting in MALDI Biotyper software version 2.0.

Based on the CCI data, a score-orientated MSP dendrogram was generated using the default setting of Biotyper 2.0, and included the 23 clinical strains and the 3 ATCC strains in the database (Figure 4). According to their mass signals and intensities, a hierarchic dendrogram clustered the 23 strains of *O. anthropi* in a single group, between 20 and 25 distance levels phylogenetically distinct from the ATCC isolates present in database. The dendrogram also revealed the presence of four subgroups, each comprising the strains clustering at less than 5 distance levels, thereby indicating a high similarity between the isolates. Strains CZ1424 and CZ1443 were grouped in the same cluster with a distance level of up to 5, as were strains CZ1429 and CZ1449. Conversely, strains CZ1523 and CZ1504 were grouped in a different cluster, at a distance level greater than 10. Strain CZ1427, which showed a 60% similarity with the other strains isolated from the same patient, was grouped at an inter-strain distance level of 10–15.

## Discussion

*O. anthropi* is an adaptable bacterial species, whose individual strains can thrive in different environments. Indeed, after its molecular characterization [11] human-associated clonal complex data appear to indicate it possesses a specialized opportunistic behaviour [3]. It is frequently isolated from contaminated medical materials/devices and specimens obtained from immunocompromised patients [3], and after the first recognized case of human disease induced by this organism [16], *O. anthropi* infections causing primary or catheter-associated bacteraemia [1,17] have been increasingly reported [4]. With this in mind, when this infection did occur in our hospital, we set out to study the identification and typing of the *O. anthropi* strains through the genomic and proteomic correlation. To our knowledge, this represents the first study on strain typing of *O. anthropi* where the use of both rep-PCR and MALDI-TOF-MS-based fingerprinting were carried out. All patients developed infection during their stay in hospital, and in our Institution no cases of infection due to *O. anthropi* had been diagnosed before. Environmental and flushing solution cultures were negative for O*. anthropi*, therefore the source of the infection strains remained unclear. Fluoroquinolone monotherapy yielded good clinical response, however blood cultures from all patients became negative only after removal of CVC.

Our results indicate that all investigated strains were highly related and that they arose from a common ancestor, strongly providing evidence for a clonal origin of the infection. Interestingly, the strains detected early on during the outbreak showed a great variability in correlation range (90%–99%), while bacteria isolated later showed a correlation higher than 99%. We can therefore speculate that *O. anthropi* is able to undergo rapid modifications, allowing bacteria to adapt to a human host. The proteomic profiles, which clustered the 23 strains in a single group, unrelated to the ATCC isolates present in the database (one of which comes from leech urine), further suggest a clonal origin of the infections. Moreover strains studied showed phylogenetically different patterns from ATCC reference strains used in all methods employed and different cluster patterns under rep-PCR fingerprinting and MALDI-TOF. A likely explanation for these differences could be that rep-PCR analysis embraces the entire bacterial chromosome, whereas the main signals reported in MALDI-TOF MS are generated from ribosomal proteins alone [18,13]. Since we studied a small number of strains, we can’t draw firm conclusions about the correlation between automated rep-PCR and MALDI-TOF for molecular typing of *Ochrobactrum anthropi*. However, both methods have demonstrated a similar sensitivity in discriminating the variability among the strains studied. Although strict comparison between PFGE and MALDI-TOF was problematic, due to the different methods involved (i.e., protein profiling for MALDI-TOF dendrogram and genetic profiling for PFGE), the tests showed a similar separation between the CZ1552 strain and the other strains. Although the results obtained by the two techniques were similar, on the whole, MALDI-TOF results were obtained much more rapidly, within a few minutes. MALDI-TOF is not only much easier and less-time consuming than PFGE, it also requires a limited amount of bacterial colonies and allows comparison at all times with the universal database.

Semi-automated rep-PCR appeared to be more discriminative than PFGE in typing the 23 *O. anthropi* strains isolated during this hospital outbreak. Both rep-PCR and MALDI-TOF MS yielded four clusters and a common ancestor, while PFGE showed the same PFGE profile in 22 isolates. In PFGE, strain CZ1552 was the odd one out, whereas rep-PCR identified strain CZ1424 as being different. These strains were found to be genetically unrelated to each other.

The marker used for the rep-PCR analysis (the region between the noncoding repetitive sequences in bacterial genomes) is less genetically stable than the one used for PFGE (the target sequence of the *SpeI* restriction enzyme). Hence, the variability shown by rep-PCR is likely to represent changes in the same clone that could not be detected by PFGE [19]. Rep-PCR analysis is a technique aimed at defining clonal relationships, and its ease of use and faster turnaround time as compared to PFGE makes it a rapid method of screening outbreaks of *O. anthropi* and therefore allows timely implementation of control measures.

## Conclusions

In conclusion, rep-PCR and MALDI-TOF MS appear to be extremely useful for evaluation of clonal relationships between isolates. The different marker (genomic vs. proteomic) evaluated, as well as the completely different techniques used increase the reliability with which isolate similarity or diversity may be assessed during a hospital outbreak. In addition, we believe that advances in the molecular typing of *Ochrobactrum anthropi* would facilitate the study on the epidemiology, prevention and control of the infections caused by this pathogen.

## Abbreviations

O. anthropi: *Ochrobactrum anthropi*; rep-PCR: Repetitive extragenic palindromic-polymerase chain reaction; MALDI-TOF: Matrix-assisted laser desorption/ionization-time-of-flight; PFGE: Pulsed-field gel electrophoresis; UPGMA: Unweighted pair group method with arithmetic mean; CVC: Central venous catheter; O. intermedium: *Ochrobactrum intermedium*; TSI: Triple Sugar Iron; HCCA: a-cyano-4-hydroxycinnamic acid; MSP: Minimum spanning trees; CCI: Composite correlation index.

## Competing interests

The study was supported by Dept of Health Sciences, “Magna Graecia” University of Catanzaro. None of the authors has a financial relationship with other people or organizations that could inappropriately influence its findings.

## Authors’ contributions

AQ participated in the design of the study, drafted the manuscript and carried out automated repetitive extragenic palindromic-polymerase chain reaction, GP carried out MALDI-TOF MS and PFGE analysis and contributed in the draft of the manuscript, ^,^ LR carried out automated repetitive extragenic palindromic-polymerase chain reaction, RP and NM carried out bacteriological cultures and identification of microorganisms, MRC participated and coordinated study on proteomic analysis, GM participated in the design and contributed in the draft and editing of the manuscript, MCL participated in the design and coordination of the study and contributed in the draft and editing of the manuscript, AF conceived the study and participated in its design and coordination. All authors read and approved the final manuscript.
